# Camera‐based independent couch height verification in radiation oncology

**DOI:** 10.1120/jacmp.v16i5.5500

**Published:** 2015-09-08

**Authors:** Martijn Kusters, Rob Louwe, Liesbeth Biemans‐van Kastel, Henk Nieuwenkamp, Rien Zahradnik, Roy Claessen, Ronald van Seters, Henk Huizenga

**Affiliations:** ^1^ Department of Radiation Oncology Radboud University Medical Center Nijmegen The Netherlands; ^2^ Department of Radiation Oncology Wellington Blood and Cancer Center Wellington New Zealand; ^3^ Panasonic Electric Works Sales Western Europe Best The Netherlands

**Keywords:** patient, setup, couch, height, radiation therapy

## Abstract

For specific radiation therapy (RT) treatments, it is advantageous to use the isocenter‐to‐couch distance (ICD) for initial patient setup.^(1)^ Since sagging of the treatment couch is not properly taken into account by the electronic readout of the treatment machine, this readout cannot be used for initial patient positioning using the isocenter‐to‐couch distance (ICD). Therefore, initial patient positioning to the prescribed ICD has been carried out using a ruler prior to each treatment fraction in our institution. However, the ruler method is laborious and logging of data is not possible. The objective of this study is to replace the ruler‐based setup of the couch height with an independent, user‐friendly, optical camera‐based method whereby the radiation technologists have to move only the couch to the correct couch height, which is visible on a display. A camera‐based independent couch height measurement system (ICHS) was developed in cooperation with Panasonic Electric Works Western Europe. Clinical data showed that the ICHS is at least as accurate as the application of a ruler to verify the ICD. The camera‐based independent couch height measurement system has been successfully implemented in seven treatment rooms, since 10 September 2012. The benefits of this system are a more streamlined workflow, reduction of human errors during initial patient setup, and logging of the actual couch height at the isocenter. Daily QA shows that the systems are stable and operate within the set 1 mm tolerance. Regular QA of the system is necessary to guarantee that the system works correctly.

PACS number: 87.55 QR

## I. INTRODUCTION

For many treatment indications, daily setup corrections are omitted in favor of off‐line correction protocols^1^, ^2^, ^3^ which are used to improve patient position. These off‐line correction protocols can effectively reduce systematic setup errors with only limited amounts of imaging dose. A reproducible and accurate initial patient setup is required, however, to effectively apply an off‐line correction protocol. In particular, when off‐line correction protocols are applied to patients who are treated on different machines during a course of radiotherapy, the varying degrees of couch sag may result in considerable setup errors.

In our institution, the ICD is used for initial patient setup during most treatments, except head and neck radiotherapy. In a study by van Lin et al.,^(4)^ the traditional laser setup method using skin markers to position the patient in the ventro–dorsal (VD) direction was compared with the couch height setup method using the ICD. The hypothesis underlying a possible benefit of using the ICD is that internal organ motion during the treatment course was small compared to the flexibility of the skin. It was concluded that the initial patient setup using the ICD significantly improved the accuracy of the patient position in the VD direction.

The sag of the treatment couch can be as large as 1 cm in extreme cases^(5)^ and is not taken into account by the electronic readout of the treatment couch. Therefore, initial patient positioning to the prescribed ICD has previously been carried out in our institution using a ruler prior to each treatment fraction, by measuring the distance between the in‐room isocenter lasers and the couch top. However, the ruler method is laborious and logging of data is not possible. The objective of this study is to replace the ruler‐based setup of the couch height with an independent, user‐friendly, optical camera‐based method where the radiation technologists have to move only the couch to the correct couch height, which is visible on a display.

## II. MATERIALS AND METHODS

**Figure 1:**
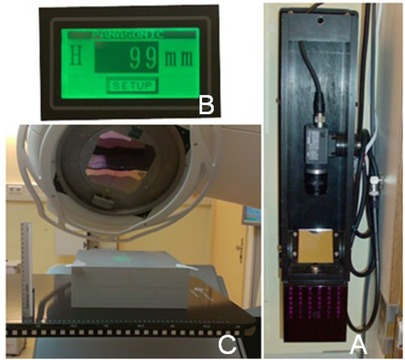
The optical parts of the ICHS: a) camera and mirror, b) digital display, and c) reference strip at the lateral side of the treatment couch. The ruler as previously applied to determine the ICD clinically is positioned on the treatment couch for illustration purposes.

The ICHS was developed in cooperation with Panasonic Electric Works Western Europe. The camera system is placed at the wall of the treatment room and a strip with a distinctive pattern is mounted at the lateral side of the couch to enable detection of the couch with infrared light. A double‐speed camera is connected to an image‐checker system containing a programmable logic controller (PLC). The PLC interprets the image of the strip in real time, calculates the couch height, and transfers the real‐time data to a digital display of the ICD in mms (Fig. 1).

Registration of the couch height for administration and analysis purposes is built into the existing protocol to avoid additional operations. An important aspect of the ICHS implementation was the improved workflow for patient setup, which now combines alignment of the patient based on the laser skin marks, movement of the couch to the correct ICD using the ICHS, and off‐line patient positioning setup corrections with the Theraview Couch Setup Assistant system (TCSA, Cablon Medical B.V., Leusden, The Netherlands). The latter system was installed previously in our department and facilitates remote couch control, as well as safe application of off‐line couch corrections. With the improved clinical workflow, the radiation technologists first change the couch height to match the displayed ICHS to the prescribed ICD. Subsequently, the radiation technologists confirm the correctness of this initial patient setup position using a dedicated pushbutton of the treatment couch. This triggers logging of the vertical position of the treatment couch to a separate log file, including the values as reported by the ICHS as well as the electronic read out of the treatment couch. In addition, activating this pushbutton will trigger the TCSA system to automatically apply the patient‐specific off‐line setup correction as defined in a dedicated database.

During installation of the system, the ICHS is calibrated against a ruler measurement using the submillimeter readout of the ICHS in service mode. During this process, the offset and scaling factor of an amplification stage in the ICHS electronics that control the view angle of the camera can be adjusted to allow measurement of ICDs between 0 and 30 cm. Calibration of the system starts by placing the strip at the center of the camera field of view and adjusting the offset. Subsequently, the scaling factor is adjusted so that the total view angle of the camera includes the treatment couch positions where the ICD equals 0 and 30 cm, respectively. After installation, commissioning of the ICHS included comparison of the logged ICHS treatment couch position and the height measured using a ruler at a broad range of treatment couch positions in all three dimensions. This was performed both with and without couch sag induced by placing a phantom on the couch top. Clinical implementation was only carried out after regular quality assurance (QA) of the system was instigated to guarantee that the system works correctly, including daily, monthly, and annual QA.

### A. Daily QA

The daily QA test of the ICHS could be combined with the daily output check of the treatment machine, because the phantom used for this output measurement is positioned by aligning the external markers on the phantom to the in‐room lasers which corresponds to a fixed ICD of 70 mm. The warning level for deviations of the ICHS readout from 70 mm is set to 1 mm, and when this tolerance is exceeded, a technician is called to further investigate and correct the system, if necessary. In addition, the ICHS measurements during the daily QA are logged by activating the dedicated TCSA pushbutton.

### B. Monthly QA

Every month a more extensive test is performed, whereby the readout of the ICHS is verified using a ruler at a range of vertical, lateral, and longitudinal positions of the treatment couch.

### C. Annual QA

Every year the optical system has to be monitored and the following are checked:

1. Brightness of the captured images,
2. Number of dead pixels within the images, due to radiation,
3. Optimal settings for the optical and digital parts like diaphragm and thresholds of the system, and
4. Verification of the calibration factor of the ICHS.

The accuracy of the ICHS in a clinical setting was assessed using the logged TCSA corrections after initial patient setup during the treatment of prostate cancer patients.

## III. RESULTS

**Figure 2:**
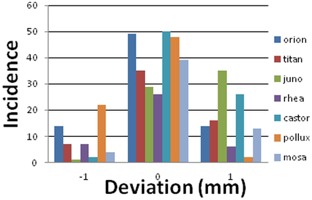
Histogram of the ICD deviations logged by the ICHS during daily QA of the camera systems at seven treatment machines from 1 January 2014 to 15 April 2014.

ICHS and ruler measurements over a broad range of treatment couch positions obtained during commissioning of the ICHS showed that the differences between ruler and ICHS measurements were always less than 0.5 mm. In addition, the actual couch height, as logged by the ICHS during daily QA tests, were analyzed to verify the inherent accuracy of the system. Figure 2 shows a histogram of the data of seven treatment machines over a period of 3.5 months. The average logged deviation of the ICHS data from the prescribed ICD of 70 mm was 0.2 mm ±0.6 mm (1 SD) and ranged from −1mm to +1 mm. It must be noted that these deviations result from: a) uncertainties of the ICHS accuracy; b) day‐to‐day interobserver variation in positioning the phantom; c) variations in the alignment of the isocenter lasers, as these are used to position the daily QA phantom; and d) deviations in the alignment and levelness of the treatment couch, as these directly induce errors in the levelness of the reflective strip and, therefore, degrade the accuracy of the ICHS measurements. Considering that multiple error sources and uncertainties contributed to the observed daily QA results, it could be concluded that the inherent accuracy of the ICHS is better than 1 mm. Furthermore, the set acceptance level of 1 mm for deviations during daily QA has been exceeded less than once a year for each system for all treatment facilities within our department. This showed that the ICHS is a reliable and robust system.

The data obtained during clinical application of the ICHS showed that the average TCSA correction in VD direction was 0.0 mm ±4.3 mm (1 SD) after initial patient setup using the ICHS. Remarkably, the clinical data obtained before implementation of the ICHS showed that the average TCSA correction in VD direction was 2.5 mm ±4.8 mm (1 SD) while using a ruler to verify the ICD. It should be noted, however, that the implementation of the ICHS coincided with the replacement of the previously applied Elekta C‐arm treatment couch tops (Elekta, Crawley, UK) by UCT(LE) couch tops (Civco Medical Solutions, Orange City, IA) because the former couch tops were worn and degraded, and the flatness and levelness were outside specification. This explains the systematic difference between the two clinical datasets obtained while either applying a ruler or the ICHS to set the ICD. This explanation was supported by the observation that the various commissioning and QA tests conducted since the replacement of the old treatment couch top consistently showed that there was no systematic difference between ICD measurements using either a ruler or the ICHS system. Finally, the clinical data confirmed that the ICHS has a very similar precision as verifying the ICD using a ruler considering that the standard deviations of the two datasets are nearly identical.

## IV. DISCUSSION & CONCLUSION

The camera‐based independent couch height measurement system has been successfully implemented in seven treatment rooms since 10 September 2012. The benefits of this system are a more streamlined workflow, reduction of human errors during initial patient setup, and logging of the actual couch height at the isocenter. Daily QA shows that the systems are stable and operate within the set 1 mm tolerance. Regular QA of the system is necessary to guarantee that the system works correctly.

## ACKNOWLEDGMENTS

We thank P. de Graaf (Dept. of Radiation Oncology, Radboud University Medical Centre) and J. Coolen (Panasonic Electric Works Western Europe, Best, The Netherlands) for their support in this project.
